# UTI-Associated Septic Cardiomyopathy: Saving the Heart in the Nick of Time

**DOI:** 10.7759/cureus.29957

**Published:** 2022-10-05

**Authors:** Saliha Erdem, Avijit Das, Rana Ismail, Hassan Makki, Arif Hakim

**Affiliations:** 1 Internal Medicine, Wayne State University School of Medicine, Detroit, USA; 2 Clinical Research, Michigan State University School of Osteopathic Medicine, Detroit, USA; 3 Pulmonary, Critical Care and Sleep Medicine, Wayne State University School of Medicine, Detroit, USA; 4 Internal Medicine/Cardiology, Beaumont Hospital, Dearborn, USA

**Keywords:** community acquired uti, severe sepsis, acute heart failure, cardiomyopathy, left ventricle

## Abstract

A patient with gram-negative sepsis developed acute global biventricular dysfunction with reduced left ventricular ejection fraction. A diagnosis of sepsis-induced cardiomyopathy (SICM) was made following the complete resolution of cardiac dysfunction. This case highlights the importance of the early diagnosis of SICM and treatment of the underlying cause.

## Introduction

Sepsis is a major health problem worldwide and is responsible for about 10% of intensive care unit admissions [1]. Septic-induced cardiomyopathy is usually underdiagnosed, with increased mortality between 70% and 90% in patients with sepsis. Its incidence ranges from 18% to 40%. The relationship between sepsis and sepsis-induced myocardial dysfunction is well-established in the current literature; however, there are insufficient clinical data defining and treating the syndrome [2]. Herein, we present a case of urinary tract infection-associated septic cardiomyopathy. Our goal is to increase the clinical awareness of this prevalent, yet underdiagnosed, entity and to further define the syndrome for early diagnosis and treatment.

## Case presentation

After obtaining patient consent, we present the case of a 51-year-old female who was brought to the Emergency Department with altered mental status and failure to thrive. On further assessment, she was noted to have diabetic ketoacidosis (DKA) and right-sided pyelonephritis, requiring intubation, mechanical ventilation, and vasopressor support. Vital signs on admission were temperature: 38.20C, blood pressure (BP): 104/60 mmHg, respiratory rate (RR): 26 breaths/min, and heart rate (HR): 114 beats/min. She was flaccid and unable to withdraw extremities in response to painful stimuli. S1 and S2 were heard in regular rhythm with no murmurs, gallop, or rub. The point of maximal impulse was not displaced and palpated at the fifth intercostal space at the midclavicular line. The remainder of the physical examination was unremarkable. Electrocardiography (EKG) revealed sinus tachycardia with an HR of 112 beats/minute and no ischemic changes. Initial troponin was elevated at 370 ng/ml. Laboratory testing was significant for lactic acid of 11.3 mmol/L, blood urea nitrogen (BUN) of 47 mg/dl, creatinine of 4.93 mg/dl (baseline Cr: 1.08mg/dl), white blood cell (WBC) count of 30x109/L, and platelet count of 225,000/ul (Table 1). Urinalysis revealed pyuria and positive leukocyte esterase. Blood cultures grew *Escherichia coli *(susceptible to ceftriaxone). Computed tomography of the abdomen-pelvis without contrast confirmed early-onset, right-sided pyelonephritis. Hence, we initiated aggressive fluid resuscitation therapy and empiric IV antibiotics for suspected sepsis and meningitis with vancomycin, and ceftriaxone along with dexamethasone and acyclovir.

**Table 1:** Laboratory values Abbreviations: WBC; white blood cell, BUN; blood urea nitrogen

The laboratory test	Admission	Day-2	Day-3	Reference range
Troponin I (ng/ml)	370	8094	2849	3-17
Lactic acid (mmol/L)	11.3	8.9	5.1	0.4-2
BUN (mg/dl)	47	64	66	7-25
Creatinine (mg/dl)	4.93	3.55	3.44	0.6-1.2
WBC (K/ml)	30	14.3	12.5	3.5-10.6
Platelet count (K/ml)	225	154	78	150-450

**Figure 1:**
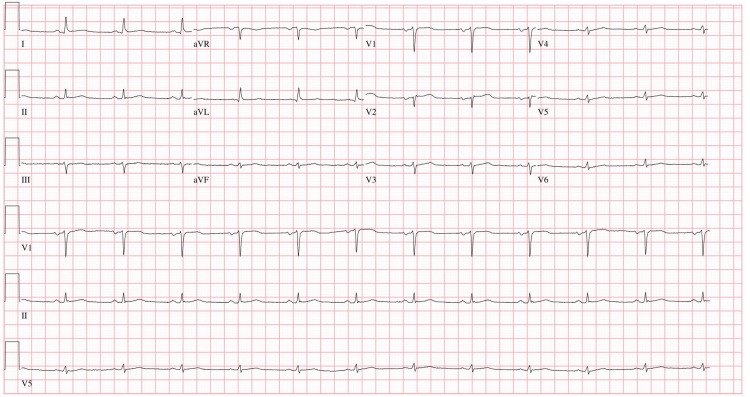
Twelve-lead EKG taken on day 2 showing sinus rhythm with an HR of 71 bpm EKG; electrocardiography, HR; heart rate

**Figure 2:**
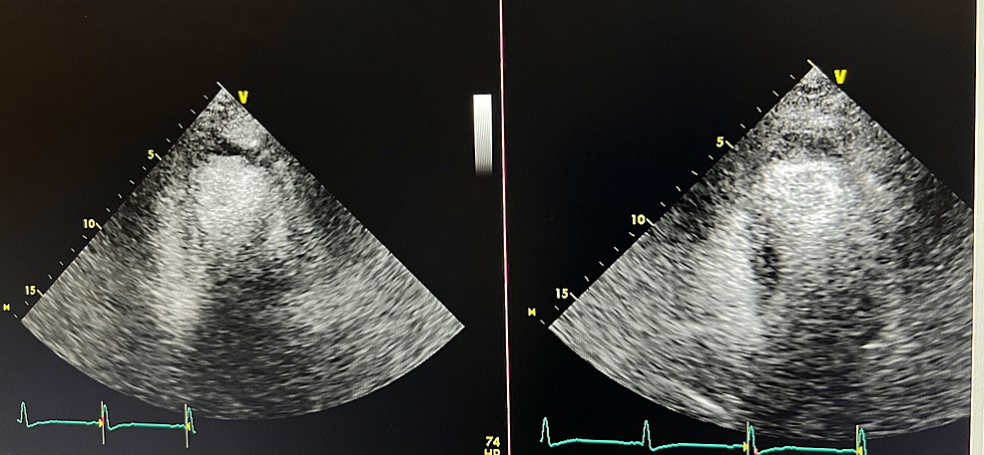
Transthoracic echocardiography four-chamber view showing reduced EF at the end of systole and diastole, respectively EF; ejection fraction

**Figure 3:**
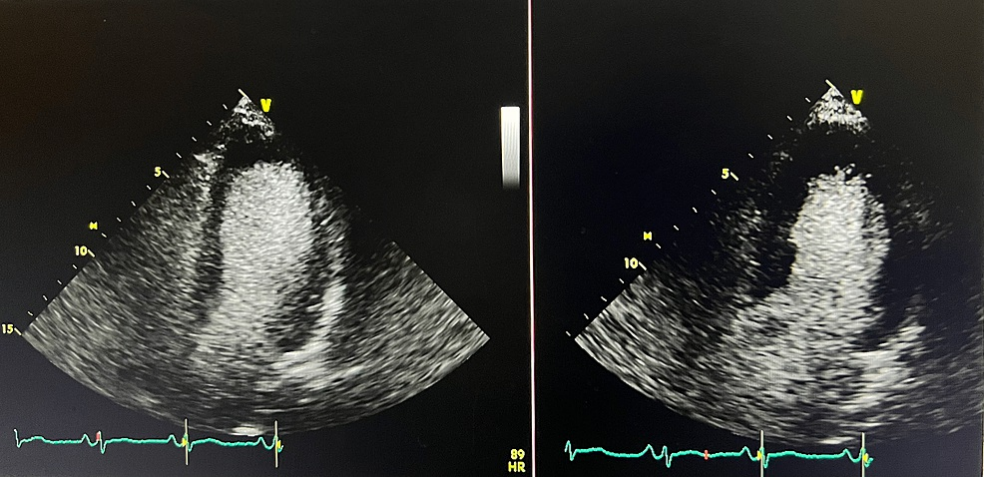
Transthoracic echocardiography four-chamber view showing normal EF at the end of systole and diastole, respectively EF; ejection fraction

On day 2, the patient exhibited nonspecific ST-segment changes on telemetry, with the 12-lead EKG revealing sinus rhythm and a heart rate of 71 beats/min, and no ischemic changes (Figure 1). Serum troponin trended up from 370 to 8094 ng/ml, and chest X-ray (CXR) was clear with no sign of cardiomegaly. Two-dimensional (2D) echocardiography demonstrated a normal-sized left ventricle with no concentric hypertrophy and mild basal septum hypertrophy. However, it showed severe global left ventricular systolic dysfunction with an estimated ejection fraction of 15% and impaired relaxation (Videos 1-2, Figure 2). Right ventricular systolic pressure (RVSP) was 20 mmHg, and there were no significant valvular abnormalities. Because of the increased risk of bleeding, we withheld IV anticoagulation, canceled cardiac catheterization, addressed the septic shock requiring antibiotics, fluid resuscitation, and vasopressor support, and treated the acute kidney injury. Cerebrospinal fluid analysis and cultures ruled out meningitis; hence, the antibiotic regimen was tailored to the treatment of pyelonephritis (vancomycin and acyclovir were discontinued, and the patient remained on ceftriaxone only). Two days later, echocardiography showed complete recovery of cardiac function with an ejection fraction of 55-60% and a normal left ventricular global contractility (Videos 3-4, Figure 3). 

**Video 1:** Transthoracic echocardiography; the parasternal long-axis view shows decreased global LV dysfunction LV; left ventricular

**Video 2:** Transthoracic echocardiography; the parasternal short axis view shows decreased global LV dysfunction LV; left ventricular

**Video 3:** Transthoracic echocardiography; the parasternal long-axis view shows resolution of LV dysfunction and impaired contractility following treatment of underlying sepsis LV; left ventricular

**Video 4:** Transthoracic echocardiography; the parasternal short axis view shows resolution of LV dysfunction and impaired contractility following treatment of underlying sepsis LV; left ventricular

Past medical history

The patient had a history of hypertension and insulin-dependent type-2-diabetes mellitus. The patient had no past medical history of coronary artery disease nor a history of surgical interventions. She reported no tobacco smoking, but daily marijuana and occasional alcohol use. No other illicit drug abuse was reported.

Differential diagnosis

The differential diagnosis of sepsis-induced cardiomyopathy is broad and includes ischemic cardiomyopathy due to acute coronary syndrome and non-ischemic causes of cardiomyopathy, including stress-induced cardiomyopathy and substance-induced cardiomyopathy.

Investigations

Upon identifying ST-segment changes on the telemetry monitor, we ordered a 12-lead EKG and serum troponin level to exclude acute coronary syndrome. The EKG revealed a sinus rhythm with a normal heart rate and a lack of any ischemic changes. However, the patient had a marked troponin elevation of 8094 ng/ml. Cardiac catheterization was deemed unsuitable due to her acute kidney injury and septic shock. A 2D echo demonstrated a normal-sized left ventricle with no concentric hypertrophy and mild basal septum hypertrophy. There was severe global left ventricular systolic function with an ejection fraction of 15% and impaired relaxation (Videos 1-2, Figure 2). The right ventricular systolic pressure was 20 mmHg, and there were no significant valvular abnormalities. The echo did not reveal any abnormalities consistent with Takotsubo cardiomyopathy (broken heart syndrome). A follow-up echo two days later showed a complete recovery of left ventricle systolic and diastolic dysfunction with an ejection fraction of 55-60%, with left ventricle global contractility returning to normal, which raised suspicion of septic cardiomyopathy (Videos 3-4, Figure 3). Follow-up troponin was 2849 ng/ml. Pro-B-type brain natriuretic peptide (BNP) was not ordered in this case.

Management

Our patient experienced septic shock due to right-sided pyelonephritis secondary to *Escherichia coli *bacteremia, for which she received early fluid resuscitation and empiric broad-spectrum antibiotics. Despite aggressive fluid resuscitation, the patient needed vasopressor support with norepinephrine to maintain a main arterial pressure (MAP) of > 65 mmHg. Antibiotics were de-escalated from broad-spectrum to IV ceftriaxone based on blood culture results and sensitivities. We initiated goal-directed medical therapy for heart failure with reduced ejection fraction after the initial Echo results. However, angiotensin-converting enzyme inhibitors and angiotensin receptor blockers were discouraged due to acute kidney injury. The follow-up echo illustrated myocardial recovery following the above treatment interventions (Videos 3-4, Figure 3). On day 3 of hospitalization, we discontinued vasopressor support, and the patient’s mental status improved. Also, her lactic level dropped to 5.1 mmol/L, her WBC count improved to 12.5, and her platelet count dropped to 78 (Table 1). The acute kidney injury was improved (Table 1).

## Discussion

Sepsis and associated septic shock account for many intensive care unit admissions worldwide and an alarming mortality rate of 40-50% [1]. Sepsis-induced cardiomyopathy, or sepsis-induced myocardial dysfunction, is a transient cardiac dysfunction recognized since the 1980s, with a prevalence of 10% to 70% among septic patients [2]. Echocardiography is the gold standard to diagnose sepsis-induced cardiomyopathy. It primarily shows acute global biventricular dysfunction with reduced left ventricular contractility that is reversible within seven to 10 days without acute coronary syndrome (ACS); however, there is no consensus on definitive diagnostic criteria [2].

Our patient underwent a septic shock from *Escherichia Coli *bacteremia requiring vasopressor support and mechanical ventilation in the intensive care unit. Initial transthoracic echocardiogram showed an estimated ejection fraction of 15 % with severe global left ventricular systolic dysfunction and mild hypertrophy involving basal septum (sigmoid septum) without left ventricular outflow tract narrowing (Videos 1-2, Figure 2). Our transthoracic echocardiogram findings ruled out Takotsubo cardiomyopathy based on the absence of apical ballooning and hyperkinesis of basal walls suggestive of Takotsubo or what is known as stress-induced cardiomyopathy [3].

Also, one study showed that sepsis-induced cardiomyopathy had higher troponin elevation at the time of diagnosis than stress-induced cardiomyopathy, although there was no suggested threshold troponin value to differentiate the two entities [3]. A thorough initial investigation of the acute coronary syndrome was performed and ruled out, given that left ventricular dysfunction was global rather than regional and that classic evolution of myocardial infarction was not seen in our serial EKGs, favoring suspicion of sepsis-induced myocardial dysfunction. The patient was deemed unstable for a left heart catheterization. A follow-up echocardiogram showed complete left ventricular recovery with a 55-60% ejection fraction (Videos 3-4, Figure 3). Moreover, the down-trending of troponin and lactic acid coincided with left ventricular recovery.

Studies revealed a higher prevalence of sepsis-induced cardiomyopathy in male patients, younger, with higher lactic acid levels and illness severity scores, and a history of heart failure [2,4]. Our patient was a relatively young female without any pre-existing heart disease; however, her illness severity score (Sequential Organ Failure Assessment (SOFA) 11, Acute Physiology and Chronic Health Evaluation (APACHE) 18) and admission lactate level were high. Although N terminal pro-brain natriuretic peptide (NT-pro BNP) elevation is a known predictive parameter for overall mortality and cardiovascular events, we failed to assess it in this patient [3].

The mechanism of cardiac dysfunction in sepsis-induced cardiomyopathy results from inflammation, mitochondrial dysfunction, abnormal calcium utilization, decreased beta-adrenergic signaling, or excess catecholamines induced by the primary insult of the infectious agent, the body’s response to sepsis, and the iatrogenic interventions [1]. Furthermore, endotoxin (lipopolysaccharide) in *Escherichia coli* plays a fundamental role in the pathogenesis of heart and kidney dysfunction in the setting of gram-negative sepsis, which might also be a contributing factor to heart and kidney impairment in this patient. Therefore, early recognition of sepsis-associated acute kidney injury and heart dysfunction may improve clinical outcomes [5].

There are limited data in the literature about the management of sepsis-induced cardiomyopathy. The treatment depends on the initial clinical suspicion and choosing the appropriate antibiotic therapy for the infection etiology. Early goal-directed therapy for septic shock with IV fluid resuscitation, broad-spectrum antibiotics and narrowing based on culture reports, and the utilization of norepinephrine for hypotension remains paramount [2]. Although there is no definitive treatment for sepsis-induced cardiomyopathy, in our case, we focused on treating the underlying septic shock with IV fluid resuscitation, antibiotics, and norepinephrine. The source of *Escherichia Coli* bacteremia was right-sided pyelonephritis. Other therapeutic modifications consist of using vasopressors other than norepinephrine, beta-blockers, levosimendan (calcium sensitizer and potassium channel activator), and mechanical support to improve cardiac output remain controversial and sometimes increase mortality [2]. As the high adrenergic state plays a role in the pathogenesis of sepsis-induced cardiomyopathy, starting beta-blockers may be beneficial to decrease myocardial oxygen demand and increase diastolic filling. However, their use in the setting of sepsis is debatable, as any negative inotropic effect could be detrimental to a patient who is relying on a hyperdynamic state [2,4].

## Conclusions

This case highlights the importance of early recognition of sepsis-induced cardiomyopathy in patients with gram-negative septicemia due to urinary tract infection. Left ventricular dysfunction attributed to sepsis-induced cardiomyopathy resolved following treatment of the underlying sepsis. Better diagnostic criteria should be adopted to recognize sepsis-induced cardiomyopathy, decrease the associated mortality, and prevent unnecessary early invasive procedures. Since there is no definitive treatment for sepsis-induced cardiomyopathy, the focus should be on treating the underlying causes.
